# Sleeve Gastrectomy Ameliorates Diabetes-Induced Cardiac Hypertrophy Correlates With the MAPK Signaling Pathway

**DOI:** 10.3389/fphys.2021.785799

**Published:** 2021-11-11

**Authors:** Qian Xu, Huanxin Ding, Songhan Li, Shuohui Dong, Linchuan Li, Bowen Shi, Mingwei Zhong, Guangyong Zhang

**Affiliations:** ^1^Department of General Surgery, Shandong Provincial Qianfoshan Hospital, Shandong University, Jinan, China; ^2^Department of General Surgery, The First Affiliated Hospital of Shandong First Medical University, Jinan, China

**Keywords:** diabetes-induced cardiac hypertrophy, sleeve gastrectomy, MAPK, ERK1/2, DUSP6

## Abstract

**Background:** Cardiac hypertrophy as a main pathological manifestation of diabetic cardiomyopathy (DCM), is a significant complication of diabetes. Bariatric surgery has been proven to relieve DCM; however, whether it can alleviate diabetes-induced cardiac hypertrophy is undefined.

**Methods:** Diabetic and obese rats were performed sleeve gastrectomy (SG) after having diabetes for 16weeks. The rats were euthanized 8weeks after SG. Metabolic parameters, heart function parameters, myocardial glucose uptake, morphometric and histological changes, and the expression level of mitogen-activated protein kinases (MAPKs) were determined and compared among the control group (CON group), diabetes mellitus group (DM group), sham operation group (SHAM group), and SG group.

**Results:** Compared with the SHAM group, the blood glucose, body weight, insulin resistance, and other metabolic parameters were significantly improved in the SG group. There was also a marked improvement in myocardial morphometric and histological parameters after SG. Furthermore, the myocardial glucose uptake and heart function were reversed after SG. Additionally, the phosphorylation of MAPKs was inhibited after SG, including p38 MAPKs, c-Jun N-terminal kinases (JNKs), and extracellular signal-regulated kinases 1/2 (ERK1/2). The expression of DUSP6, which dephosphorylates ERK1/2, was upregulated after SG. These findings suggest that SG ameliorated diabetes-induced cardiac hypertrophy correlates with the MAPK signaling pathway.

**Conclusion:** These results showed that diabetes-induced cardiac hypertrophy was ameliorated after SG was closely related to the inhibition of the MAPK signaling pathway and upregulation of DUSP6. Therefore, this study provides a novel strategy for treating diabetes-induced cardiac hypertrophy.

## Introduction

During the past three decades, the number of people with diabetes mellitus has increased fourfold (Gibb and Hill, 2018). Diabetes and its complications, including diabetic cardiomyopathy (DCM), pose a serious threat to global health and place a great burden on global health care (Holman et al., 2015; Zimmet, 2017). DCM substantially increases a person’s risk of heart failure (Kannel et al., 1974; de Simone et al., 2010); among hospitalized patients with heart failure, approximately 44% have diabetes mellitus (Echouffo-Tcheugui et al., 2016). Cardiac hypertrophy and myocardial fibrosis are the main pathological features of DCM, with a significant association with death in DCM patients (Kobayashi and Liang, 2015; Jia et al., 2018). Unfortunately, there is no reliable strategy for treating this disease.

Initially, bariatric surgery was only used to treat morbid obesity, but we have found that it also has some benefits for the treatment of type 2 diabetes mellitus (T2DM) and its complications (Affinati et al., 2019; Ruze et al., 2020). Presently, sleeve gastrectomy (SG) and Roux-en-Y gastric bypass (RYGB) are the most common bariatric surgeries undertaken globally, accounting for 45.9 and 39.6%, respectively (Angrisani et al., 2017; Pucci and Batterham, 2019). Nowadays, multiple studies have demonstrated that bariatric surgery improved hyperglycemia and reduced body weight (Affinati et al., 2019; Arterburn et al., 2020; Docherty and le Roux, 2020). Markedly, bariatric surgery significantly alleviates T2DM and improves its complications, compared with traditional medical treatment (Nguyen and Varela, 2017). Huang et al. have shown that the bariatric surgery improved DCM and diabetes-induced cardiac hypertrophy clearly (Huang et al., 2018), yet the specific mechanism is unclear.

The mitogen-activated protein kinase (MAPK) signaling pathway has been demonstrated to play a key role in pathological cardiac hypertrophy by multiple studies (Ba et al., 2019; Liao et al., 2019). As a highly conserved signaling pathway, this pathway is widely found in mammals (Cargnello and Roux, 2011). The conventional MAPK signaling family is mainly composed of three pathways, the p38 MAPKs, the c-Jun N-terminal kinases (JNKs), and the extracellular signal-regulated kinases 1/2 (ERK1/2; Siti et al., 2021). The biological responses of MAPKs depend on their phosphorylation level (Liu et al., 2016). Chang et al. (2011) and Shen et al. (2011) have reported that these three MAPKs were upregulated in glucose-induced cardiomyocytes and in streptozotocin (STZ)-induced diabetic rats. A strong link between MAPK signaling pathway and cardiac hypertrophy has been reported (Singh et al., 2017). Among these three MAPKs, JNK and p38 attenuate cardiac hypertrophy by regulating pro-inflammation and stress, whereas ERK1/2 regulates cell growth, differentiation, and proliferation (Mutlak and Kehat, 2021). The dephosphorylation of MAPKs is achieved *via* dual-specificity phosphatases (DUSPs; Liu and Molkentin, 2016). Currently, 13 DUSPs have been characterized, among them, DUSP1 has been reported to downregulate p38, JNK, and ERK1/2 in transgenic mice (Bueno et al., 2001; Liu et al., 2016). Consistently, a DUSP1 and DUSP4 double-null mutant led to enhancement of p38 phosphorylation, which in turn reduced the survival rate (Auger-Messier et al., 2013). DUSP6 has been reported to inhibit ERK1/2 to improve cardiac hypertrophy (Ramkissoon et al., 2019). Thus, we concluded that MAPK signaling pathways and DUSPs play a vital role in diabetes-induced cardiac hypertrophy.

Our previous study has demonstrated that cardiac hypertrophy was ameliorated after bariatric surgery in diabetic rats (Huang et al., 2019). However, whether the amelioration of cardiac hypertrophy by SG is related to MAPK signaling pathways and DUSPs is unknown. In this study, we explored this using a high-fat diet (HFD)-STZ-T2DM rat model.

## Materials and Methods

### Animals

**Figure 1:**
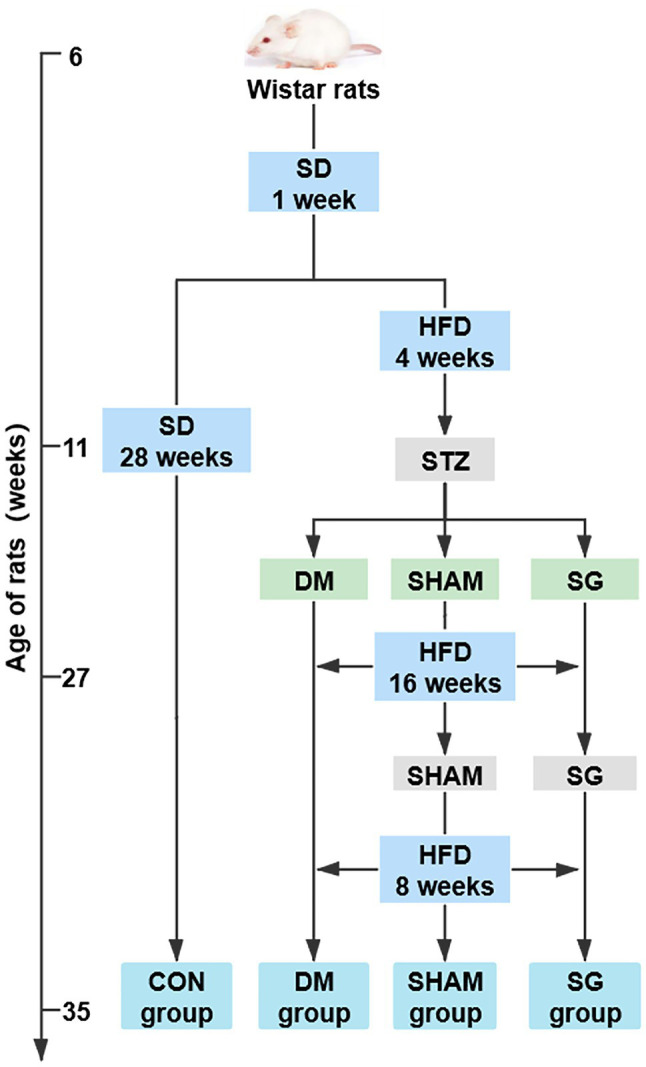
The flow chart of animal experiments. *N*=10 for per groups. SD, standard diet; HFD, high-fat diet; STZ, streptozotocin; CON, control; DM, diabetes mellitus; SHAM, sham operation; SG, sleeve gastrectomy.

Eighty 4-week-old male Wistar rats (80–110g; Vital River Laboratory Animal Technology Co., Ltd., Beijing, China) were housed in the animal laboratory of Shandong Provincial Qianfoshan Hospital of Shandong University in specific pathogen-free housing conditions at 20–26°C and 50–60% humidity. The animal study was reviewed and approved by approved by the Institutional Animal Care and Use Committee of Shandong Provincial Qianfoshan Hospital of Shandong University. All animals were adaptively fed a standard diet for 1week (SD; 15% of the calories were fat, Laboratory Animal Center of Shandong University, Jinan, China) and then randomly divided into the following four groups (Figure 1):

(1) Control group (CON group; *n*=15): fed a SD during the entire study. (2) Diabetes mellitus group (DM group; *n*=18): received a single intraperitoneal STZ (Sigma-Aldrich, St. Louis, MO, United States) injection at 33mg/kg body weight after a 4-week HFD (40% of the calories were fat; Jiangsu Xietong Pharmaceutical Bio-engineering Co., Ltd., Nanjing, China) followed by a 12-h fast. STZ was dissolved in 0.1mol/L sodium citrate buffer (pH=4.5; Beijing Solarbio Science & Technology Co., Ltd., Beijing, China) to 0.1ml/mg. Three days after the STZ injection, the random blood glucose was measured three consecutive times (≥ 16.7mmol/L was considered a successful model of diabetic rats; Zhang et al., 2020). (3) Sham operation group (SHAM group, *n*=20): treated like the DM group, but a sham operation was performed 16weeks after the STZ injection. The rats continued to be fed an HFD for 8weeks after the operation and were then euthanized. (4) Sleeve gastrectomy group (SG group, *n*=27): treated like the DM group, but the SG was performed 16weeks after the STZ injection. The rats continued to be fed an HFD for 8weeks after the operation and were then euthanized.

After the experiments, all rats in the CON group survived, five rats in the DM group died of hyperglycemia, and two and six rats in the SHAM group died of hyperglycemia and infection, respectively. In the SG group, two rats died of hypoglycemia, four rats died of intestinal obstruction, three rats died of infection, and three rats died of gastric leakage. Namely, 15, 13, 12, and 15 rats survived in the CON, DM, SHAM, and SG groups, respectively. In addition, there were 2, 0, 2, and 2 rats in CON group, DM group, SHAM group, and SG group, respectively, which were defined as outliers by Grubbs’ test. Thus, 13, 13, 10, and 13 rats in the CON, DM, SHAM, and SG groups could be included in this study, respectively. To make the sample size of each group equal, 5, 3, 2, and 5 rats from the CON, DM, SHAM, and SG groups including outliers were randomly euthanatized by injecting an overdose of 10% chloral hydrate (6ml/kg). Consequently, each group had 10 rats.

### Surgical Procedures

Sleeve gastrectomy: SG was performed as previously reported (Bruinsma et al., 2015). All rats fasted for 12h before operation and were then anesthetized with 2% isoflurane gas. First, a 4cm incision in the middle of the upper abdomen was made. Then, the stomach was be dissociated until the gastric blood vessels and tissue structure were clearly distinguished. The blood vessels of the gastric fundus and the greater gastric curvature were ligated by 7–0 lines (Ningbo Cheng-He Microsurgical Instruments Factory, Ningbo, China) and then interrupted after successful ligation. Next, a 0.5cm incision parallel to the greater gastric curvature was made at the gastric fundus to remove the stomach contents. The gastric fundus and a large portion of the gastric body on the greater gastric curvature were removed, and subsequently, the remaining stomach was sutured with a 5–0 line (Ningbo Cheng-He Microsurgical Instruments Factory). The anatomical position of the abdominal cavity was restored after confirming no active bleeding. Finally, the abdominal cavity was closed layer by layer with 3–0 lines (Ningbo Cheng-He Microsurgical Instruments Factory).

Sham operation: This operation was the same as the above SG procedure before blocking the gastric blood vessels. There were no other interventions, except for exposing abdominal organs, such as the stomach, small intestine, and liver. Additionally, to maintain the same degree of stress as the SG group, we prolonged the operative and anesthesia time in line with the SG group.

### Physiological Parameters

The body weight of all the rats was measured before the operation and 2, 4, 6, and 8weeks after the operation. The food intake was measured at the same time points as the body weight. After euthanasia, the heart weight (HW) and tibia length (TL) of the rats were measured and the HW/TL ratio was calculated and analyzed.

### Blood Chemistry

The fasting blood glucose (FBG) was measured by a glucometer (Roche One Touch Ultra, Lifescan, Johnson & Johnson, Milpitas, CA, United States) after a 12-h fast at the same time points as the body weight was measured. Blood was collected from the tail vein of anesthetized rats and immediately centrifuged for 8min at 3000rpm, and then, the upper serum was collected and stored in a frozen tube at −80°C. The serum insulin was measured by an ELISA kit (EZRMI-13K, Millipore, Darmstadt, Germany). Finally, the homeostasis model assessment of insulin resistance (HOMA-IR) was calculated to estimate the degree of insulin resistance. The calculation formula was: HOMA-IR=fasting serum insulin (mIU/L)×FBG (mmol/L) /22.5.

### Oral Glucose Tolerance Test and Insulin Tolerance Test

The oral glucose tolerance test (OGTT) was performed after a 12-h fast at the same time points as body weight was measured. Rats were administered with 1g/kg glucose by oral gavage, and then, their blood glucose was measured after 0, 10, 30, 60, and 120min. The area under the curve (AUC) of the OGTT (AUC_OGTT_) was calculated by the trapezoidal method.

Two days after the OGTT, the insulin tolerance test (ITT) was performed after a 12-h fast. Rats were administered with 0.5IU/kg insulin (Gansulin 40R, Tonghua Dongbao Pharmaceutical Co., Ltd., Tonghua, China) by intraperitoneal injection, and then, their blood glucose was measured after 0, 10, 30, 60, and 120min. The AUC of the ITT (AUC_ITT_) was calculated by the trapezoidal method.

### Echocardiographic Evaluation

Echocardiography was performed after the 12-h fast and before the euthanasia of rats with a Vevo 2,100 imaging system (VisualSonics, Toronto, Canada). Rats were anesthetized with 2% isoflurane. Doppler mode and M-mode imaging were performed to evaluate the cardiac structure. Furthermore, left ventricular end-diastolic diameter, left ventricular end-systolic diameter, ejection fraction, and fractional shortening were analyzed to evaluate the function of the left ventricle.

### Positron-Emission Tomography Scan and Image Processing

Positron-emission tomography (PET) scan was performed following a 12-h fast and before the euthanasia of rats, which were anesthetized with 2% isoflurane. After intravenous injection of 800μCi (29.6MBq) 18F-FDG, the entire rat body was continuously scanned with a PET scanner (Metis 1800, Madic Technology Co., Ltd., Linyi, China) for 60min. The PET images recorded show three dimensions: coronal, sagittal, and transverse. Additionally, the average standard uptake value (SUV_Mean_) was analyzed by PMOD 4.1 software (PMOD Technologies LLC., Zurich, Switzerland) to evaluate the myocardial glucose uptake of the rats.

### Morphometric and Histological Analysis

The heart tissue was fixed with 4% paraformaldehyde, embedded in paraffin, and sectioned into 5μm sections. The paraffin sections were stained with hematoxylin and eosin (H&E; G1003, Servicebio, Wuhan, China) to evaluate the heart structure. Furthermore, to determine collagen deposition, Sirius Red (G1018, Servicebio, Wuhan, China) and Masson (G1006, Servicebio, Wuhan, China) staining were performed on the 5μm paraffin heart tissue sections. The collagen volume fraction (CVF) was calculated by ImageJ software (National Institutes of Health, Bethesda, MD, United States) and used as a quantitative measure of collagen deposition. To determine the area of cardiac myocytes (CMs), paraffin sections of transversely cut muscle fibers were stained with wheat germ agglutinin (WGA; L4895, Sigma-Aldrich, St. Louis, MO, United States). ImageJ software was used to calculate the CM area. Oil Red O staining assessed myocardial lipid accumulation. The fresh tissue frozen sections were reheated and dried and then fixed it in the fixative solution for15 min. Oil Red solution (G1016, Servicebio, Wuhan, China) was used to stain the frozen sections. And then immersed them into isopropanol for differentiation. After stained by hematoxylin (G1004, Servicebio, Wuhan, China), followed by three washes. Finally, the sections with glycerin gelatin. All the sections were made into digital sliders by a Pannoramic Digital Slide Scanners (Pannoramic DESK, P-MIDI, P250, and P1000, 3DHISTECH Ltd., Budapest, Hungary).

### Quantitative Real-Time PCR

Total RNA was extracted from frozen heart tissue by TRIzol reagent (G3013, Servicebio, Wuhan, China). Single-strand complementary DNA (cDNA) was synthesized by ReverTra Ace qPCR RT Kit (FSQ-101, TOYOBO Co., Ltd., Osaka, Japan). For quantitative real-time PCR, the SYBR Green Realtime PCR Master Mix (QPK-201, TOYOBO Co., Ltd.) was used following the manufacturer’s instructions. The total PCR reaction volume was 20μl, containing 0.8μl of each primer. The target-specific primers were designed by Sangon Biotech (Shanghai, China), and the specific sequences are listed in Table 1.

**Table 1:** Specific sequences of primers used in real-time PCR.

Gene	Primer sequence, 5ʹ-3ʹ	Gene ID
*Anp*	F: GAGCGAGCAGACCGATGAAGC R: TCCATCTCTCTGAGACGGGTTGAC	24602
*β-mhc*	F: CACCGCCTCCTCCACCTCTG R: CCAGAACACCAGCCTCATCAACC	29557
*Col1a1*	F: TGTTGGTCCTGCTGGCAAGAATG R: GTCACCTTGTTCGCCTGTCTCAC	29393
*Col3a1*	F: AGTCGGAGGAATGGGTGGCTATC R: CAGGAGATCCAGGATGTCCAGAGG	84032
*Col4a5*	F: CATACAAGGTGTGGCGGGAAATCC R: TCCTGGCTGGCTGATGGTCTG	363457
*Col9a1*	F: GAGCCAGGAAGACAAGGACACAAG R: CCAACTATGCCAGTGATGCCTCTC	305104
*Dusp6*	F: CGGTGACAGTGGCTTACCTTATGC R: TGAAGTTGAAGTTGGGAGAGATGTTGG	116663
*β-actin*	F: TGCTATGTTGCCCTAGACTTCG R: GTTGGCATAGAGGTCTTTACGG	81822

F, forward and R, reverse.

### Immunohistochemistry

Paraffin-embedded heart tissue was sectioned into 5μm sections. The paraffin sections were deparaffinized and rehydrated in xylol and alcohol. The antigen was retrieved in a microwave oven with citrate antigen retrieval solution (ZLI 9064, ZSGB-BIO, Beijing, China) after three washes with phosphate-buffered saline (PBS, G0002-2L, Servicebio). Sections were incubated overnight at 4°C with primary antibodies (Collagen I, 1:500, ab270993, Abcam, Cambridge, MA, United States; Collagen III, 1:200, ab7778, Abcam; DUSP6, 1:100, ET1602-18, Huabio, China; p-ERK1/2, 1:200, 4,370T, Cell Signaling Technology, Danvers, MA, United States), followed by three washes with PBS. Sections were then incubated with a universal two-step detection kit (PV-9000, ZSGB-BIO) following the manufacturer’s instructions. After three washes with PBS, the sections were stained with diamino-benzidine (DAB, ZLI-9018, ZSGB-BIO) and hematoxylin and then dehydrated, and transparency were performed. Finally, the sections were made into digital slides with Pannoramic Digital Slide Scanners (Pannoramic DESK, P-MIDI, P250, and P1000, 3DHISTECH Ltd.).

### Western Blot Analysis

Total protein was extracted from frozen heart tissue using a Minute™ muscle tissue total protein extraction kit (SA-06-MS, Invent Biotechnologies, Inc., United States) following the manufacturer’s instructions. Protein samples were quantified using a BCA Protein Assay Kit (E-BC-K318-M, Elabscience, Wuhan, China). Protein samples were resolved on 10% SDS-PAGE gels (PG212, EpiZyme, Shanghai, China) and transferred onto polyvinylidene fluoride membranes (Millipore, Burlington, MA, United States). The membranes were blocked with 5% fat-free milk and incubated overnight at 4°C with primary antibodies (ANP, 1:1000, ab209232, Abcam, United States; β-MHC, 1:1000, 22280-1-AP, Proteintech, China; p38, 1:1000, ET1702-65, Huabio, China; JNK, 1:1000, RT1550, Huabio, China; ERK1/2, 1:1000, 67170-1-Ig, Proteintech, China; p-p38, 1:1000, ER1903-01, Huabio, China; p-JNK, 1:1000, 4668S, Cell Signaling Technology, USA; p-ERK1/2, 1:1000, 4370T, Cell Signaling Technology, United States; DUSP 6, 1:1000, ET1602-18, Huabio, China; β-ACTIN, 1:1000, ab8226, Abcam, United States). Then, the membranes were washed and incubated with secondary antibodies (Goat anti-Mouse IgG, 1:10000, ab216776, Abcam; Goat Anti-Rabbit IgG, 1:10000, ab6721, Abcam). The protein bands were visualized by ECL (Millipore) and quantified using ImageJ software (National Institutes of Health).

### Statistical Analysis

Data were analyzed using Graph Pad Prism 8.0 (GraphPad software, San Diego, CA, United States) and intergroup comparisons were performed with one-way ANOVA followed by Tukey’s multiple comparisons test. Statistical outliers were determined using the Grubbs’ test. *p*<0.05 was considered statistically significant. Data are presented as the mean±SEM.

## Results

### SG Significantly Improves Metabolic Parameters in Diabetic Obese Rats

**Figure 2:**
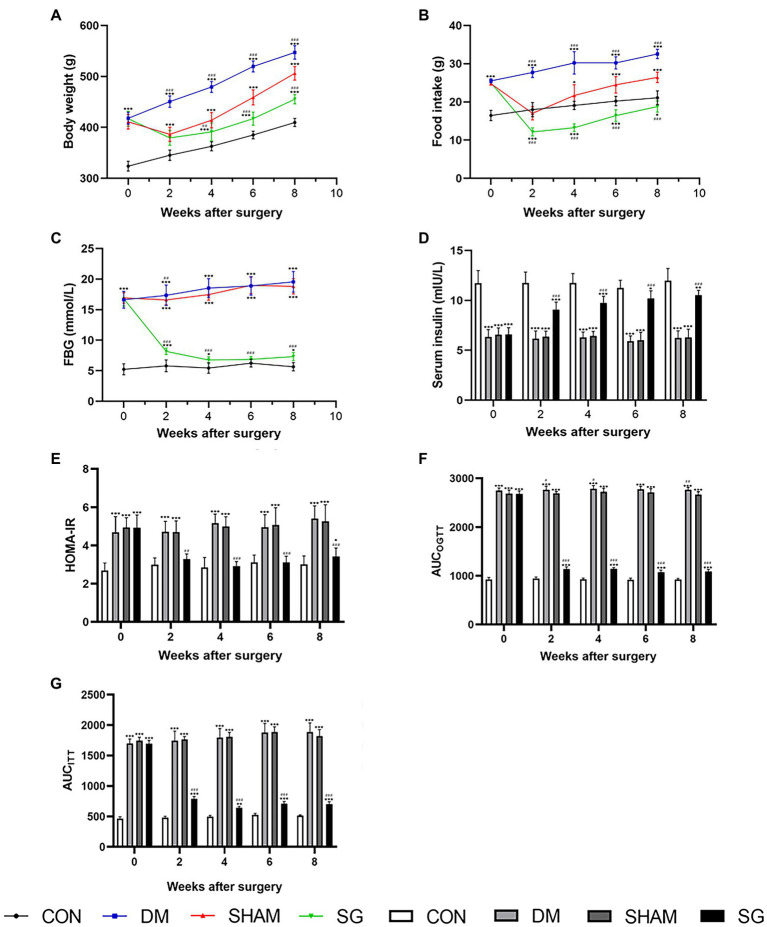
The effect of SG on the metabolic parameters. **(A)** Body weight, **(B)** food intake, **(C)** FBG, **(D)** serum insulin, **(E)** HOMA-IR, **(F)** AUC_OGTT_, **(G)** AUC_ITT_ before and after operation. Data are expressed as means±SEM for *n*=10 per groups. ^*^p<0.05 vs. CON group, ^**^p<0.001 vs. CON group, ^***^p<0.0001 vs. CON group; ^#^*p*<0.05 vs. SHAM group, ^##^*p*<0.001 vs. SHAM group, ^###^*p*<0.0001 vs. SHAM group. FBG, fasting blood glucose; HOMA-IR, homeostasis model assessment of insulin resistance; AUC_OGTT_, the area under the curve of the oral glucose tolerance test; AUC_ITT_, the area under the curve of the insulin tolerance test; CON, control; DM, diabetes mellitus; SHAM, sham operation; SG, sleeve gastrectomy.

The metabolic abnormalities in diabetic obese rats were significantly improved in the SG group (Figure 2). The results showed that the body weight and food intake of the DM group were significantly higher than those of the CON group, whereas SG significantly reduced these parameters (Figures 2A,B). Furthermore, compared with the SHAM group, the SG group had a lower level of FBG from 2weeks after the operation (Figure 2C). In addition to the change in FBG and the serum insulin level, we found that the insulin resistance of the SG group was significantly improved compared with that of the SHAM group as assessed by HOMA-IR (Figures 2C–E). Consistent with the above results, analysis of AUC_OGTT_ and AUC_ITT_ demonstrated a significant improvement in insulin resistance in the SG group (Figures 2F,G). All these data above collectively showed that SG can significantly improve the metabolic parameters of diabetic obese rats.

### SG Significantly Reverses Heart Dysfunction in Diabetic Obese Rats

**Figure 3:**
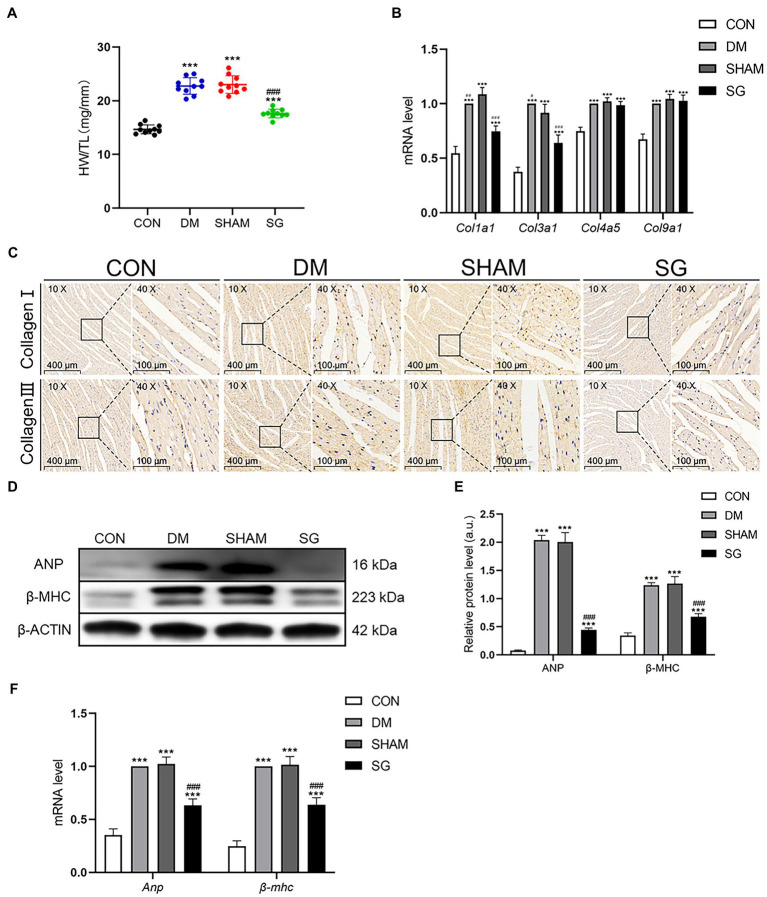
The positive effect of SG on the heart basic parameters and the expression of collagen. **(A)** HW/TL was measured. **(B)** Heart mRNA levels of *Col1a1*, *Col3a1*, *Col4a5*, and *Col9a1* were measured. **(C)** The representative immunohistochemical staining of collagen I and collagen III (magnification 10×, scale bars represent 400μm; magnification 40×, scale bars represent 100μm). Brown staining is considered positive. **(D)** The representative bands of ANP and β-MHC and **(E)** relative protein level of ANP and β-MHC were quantitatively analyzed. **(F)** Heart mRNA levels of *Anp* and *β-mhc* were measured. Data are expressed as means±SEM for *n*=10 per groups. ^*^p<0.05 vs. CON group, ^**^p<0.001 vs. CON group, ^***^p<0.0001 vs. CON group; ^#^*p*<0.05 vs. SHAM group, ^##^*p*<0.001 vs. SHAM group, ^###^*p*<0.0001 vs. SHAM group. HW/TL, heart weight/tibial length; CON, control; DM, diabetes mellitus; SHAM, sham operation; SG, sleeve gastrectomy.

Cardiac hypertrophy was evidenced by increased HW/TL in the DM group compared with the CON group. However, the SG group had a lower HW/TL compared with the SHAM group (Figure 3A). Analyzing the mRNA level of collagen genes demonstrated that the expression of *Col1a1* and *Col3a1* in the DM group was higher than that in the CON group (Figure 3B). Notably, SG significantly reduced the expression of these genes compared with that in the SHAM group, yet there was no significant difference in the expression of *Col4a5* and *Col9a1* among these four groups (Figure 3B). Furthermore, immunohistochemistry showed that the expression intensity and distribution of collagen I and collagen III in the DM group were significantly higher than those in the CON group (Figure 3C). Furthermore, the SG group showed lower expression intensity and distribution of collagen I and collagen III compared with that in the SHAM group, but it did not decrease to the level of the CON group (Figure 3C). With respect to natriuretic peptide A (ANP) and cardiac muscle myosin heavy chain β isoform (β-MHC), the DM group had a higher expression of both, compared with the CON group (Figures 3D,E). Additionally, SG significantly decreased this expression, suggesting that the cardiac hypertrophy in the SG group was improved (Figures 3D,E). Similarly, the changes in the mRNA levels of *Anp* and *β-mhc* confirmed the above results (Figure 3F).

**Figure 4:**
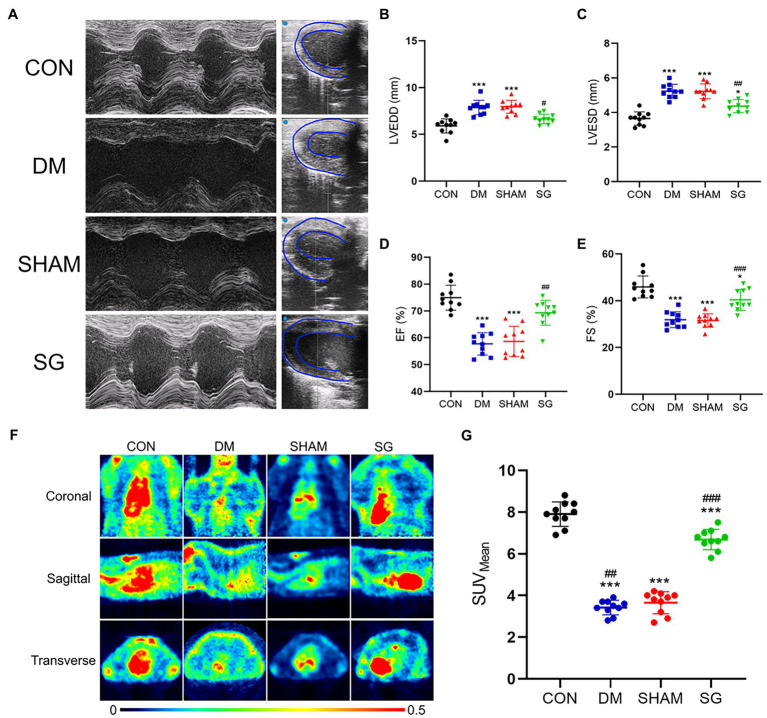
The beneficial effect of SG on the heart function and myocardial glucose uptake. **(A)** The representative images of echocardiography, and the **(B)** LVEDD, **(C)** LVESD, **(D)** EF, **(E)** FS were measured and analyzed. **(F)** The representative heart PET images of the rats and **(G)** comparisons of the myocardial glucose uptake through SUV_Mean_. Data are expressed as means±SEM for *n*=10 per groups. ^*^p<0.05 vs. CON group, ^**^p<0.001 vs. CON group, ^***^p<0.0001 vs. CON group; ^#^*p*<0.05 vs. SHAM group, ^##^*p*<0.001 vs. SHAM group, ^###^*p*<0.0001 vs. SHAM group. PET, positron-emission tomography; LVEDD, left ventricular end-diastolic diameter; LVESD, left ventricular end-systolic diameter; EF, ejection fraction; FS, fractional shortening; SUV_Mean_, the average standard uptake value; CON, control; DM, diabetes mellitus; SHAM, sham operation; SG, sleeve gastrectomy.

Moreover, echocardiography, an important method for evaluating heart function, showed that SG significantly improved the heart function and left ventricular wall thickness of diabetic obese rats (Figure 4A). The SG group showed smaller left ventricular end-diastolic diameter and left ventricular end-systolic diameter, approximately 2mm and 1mm reduction, respectively, compared with the SHAM group (Figures 4B,C). Furthermore, SG significantly elevated the ratio of ejection fraction and fractional shortening, by approximately 20 and 10%, respectively (Figures 4D,E).

All these results suggest that SG improved the degree of myocardial fibrosis and diabetes-induced cardiac hypertrophy, and it had a positive effect on the heart function impaired by DM.

### SG Significantly Improves Myocardial Glucose Uptake in Diabetic Obese Rats

Multiple studies have shown that DM can result in obstruction of myocardial glucose uptake (Steneberg et al., 2018). Thus, we evaluated myocardial glucose uptake by PET scans (Figure 4F). The SUV_Mean_ of the CON group and SG group was significantly higher than that of the DM group and SHAM group, respectively; however, the SUV_Mean_ of the SG group did not reach the level of the CON group (Figure 4G). These results suggest that SG improved the diabetes-induced obstruction of myocardial glucose uptake.

### SG Significantly Improves Myocardial Morphology and Histopathology

**Figure 5:**
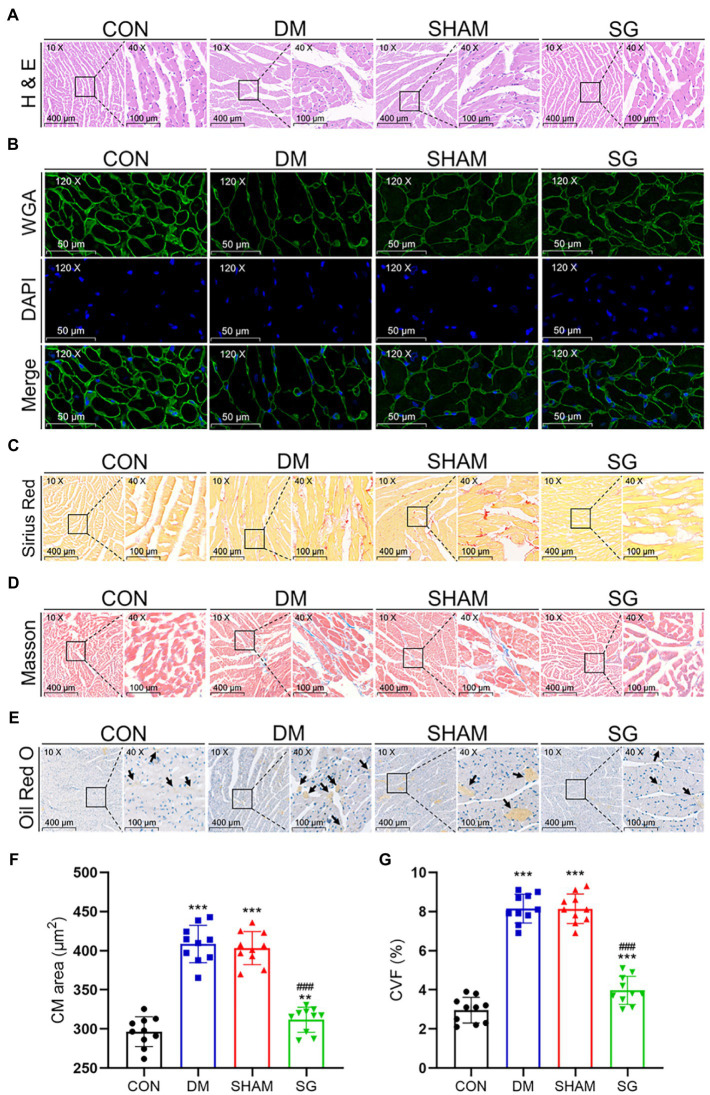
The reversal effect of SG on the heart morphometric and histological. The representative **(A)** H&E, **(B)** WGA, **(C)** Sirius Red, and **(D)** Masson stainings of heart tissue. **(E)** The Oil Red O staining of heart tissue, and arrows show lipid droplets (magnification 10×, scale bars represent 400μm; magnification 40×, scale bars represent 100μm; magnification 120×, scale bars represent 50μm). The **(F)** CM area and **(G)** CVF were calculated and analyzed. Data are expressed as means±SEM for *n*=10 per groups. ^*^p<0.05 vs. CON group, ^**^p<0.001 vs. CON group, ^***^p<0.0001 vs. CON group; ^#^*p*<0.05 vs. SHAM group, ^##^*p*<0.001 vs. SHAM group, *^###^p*<0.0001 vs. SHAM group. H&E, hematoxylin and eosin; WGA, wheat germ agglutinin; CM, cardiac myocyte; CVF, collagen volume fraction; CON, control; DM, diabetes mellitus; SHAM, sham operation; SG, sleeve gastrectomy.

Many studies have reported that diabetes causes changes in myocardial histomorphology (Sassi et al., 2017; Pabel et al., 2018). Compared with the CON group, the CMs in the DM group were disordered, the myocardial fibers were fragmented and broken, and the cytoplasmic distribution was uneven. Notably, SG significantly ameliorated these changes (Figure 5A). To evaluate CM size, WGA staining was used to analyze the CM area. The results showed that DM led to larger CMs, whereas in the SG group, the area of CMs was significantly smaller than that in the SHAM group (Figures 5B,F). Myocardial fibrosis, a significant feature of cardiac hypertrophy, was evaluated by the CVF, which was determined by Sirius Red staining and Masson staining (Figures 5C,D). The SG group showed a significantly reduction in CVF, compared with the SHAM group (Figure 5G). Oil Red O staining was performed to evaluate the lipid accumulation. As a result, compared with the SHAM group, SG group showed a significantly reduction in lipid accumulation (Figure 5E). Namely, SG reduced the size of CMs and improved their arrangement and the cytoplasmic distribution. Furthermore, it significantly improved myocardial lipid accumulation and fibrosis.

### Effects of SG on MAPK Signaling Pathways

**Figure 6:**
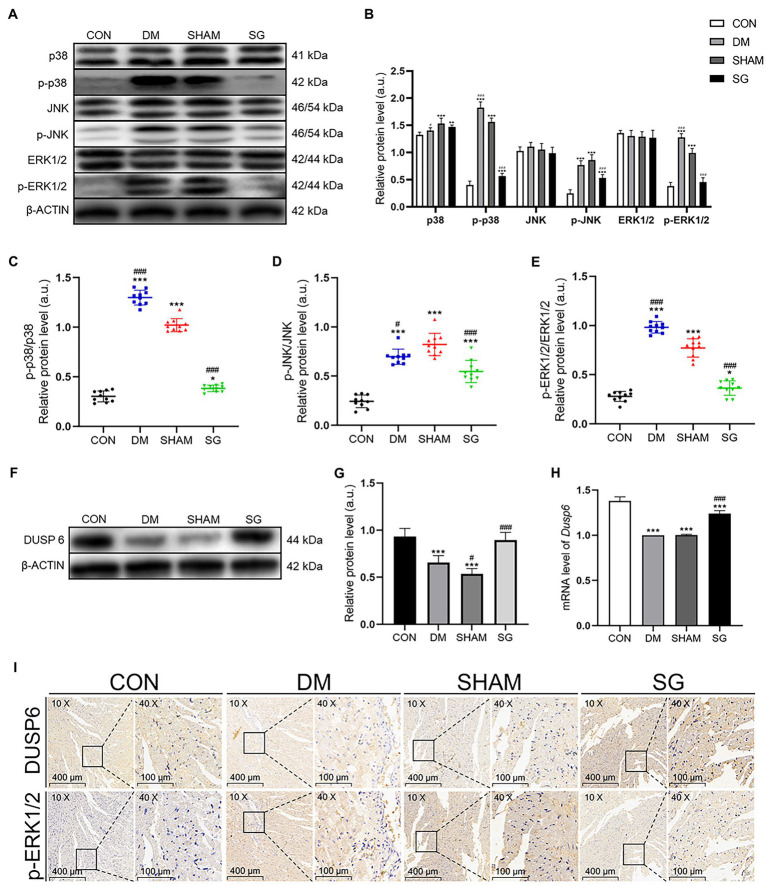
The effect of SG on MAPK signaling pathway and the expression of DUSP6. **(A)** The representative bands of p38, p-p38, JNK, p-JNK, ERK1/2, and p-ERK1/2, and **(B)** relative protein level of p38, p-p38, JNK, p-JNK, ERK1/2, and p-ERK1/2 were quantitatively analyzed. **(C–E)** The expression ratio of p-p38 over total p38, p-JNK over total JNK, and p-ERK1/2 over total ERK1/2, respectively. **(F)** The representative bands of DUSP6 and **(G)** relative protein level of DUSP6 were quantitatively analyzed. **(H)** Cardiac mRNA level of *Dusp6* was measured. **(I)** The representative immunohistochemical staining of DUSP6 and p-ERK1/2 (magnification 10×, scale bars represent 400μm; magnification 40×, scale bars represent 100μm). Brown staining is considered positive. Data are expressed as means±SEM for *n*=10 per groups. ^*^p<0.05 vs. CON group, ^**^p<0.001 vs. CON group, ^***^p<0.0001 vs. CON group; ^#^*p*<0.05 vs. SHAM group, ^##^*p*<0.001 vs. SHAM group, ^###^*p*<0.0001 vs. SHAM group. CON, control; DM, diabetes mellitus; SHAM, sham operation; SG, sleeve gastrectomy.

Based on previous studies showing that the activation of MAPK signaling pathways resulted in cardiac hypertrophy (Singh et al., 2017), we investigated the effect of SG on these pathways. Western blot was performed to analyze the expression of MAPKs. The myocardial expression of p-p38, p-JNK, and p-ERK1/2 was significantly higher in the DM group and SHAM group, compared with the CON group, but these three MAPKs were significantly downregulated after SG (Figures 6A,B). The results of the ratio of p-p38 to total p38, p-JNK to total JNK, and p-ERK1/2 to total ERK1/2 were consistent with the above result (Figures 6C–E). Taken together, we deduced that SG ameliorated diabetes-induced cardiac hypertrophy, at least partly, associated with inhibition of MAPK signaling pathways.

### Effects of SG on the Expression of DUSP6

DUSPs specifically dephosphorylate and inactivate MAPKs (Liu and Molkentin, 2016). ERK1/2 plays a key role in cell growth, differentiation, and proliferation (Mutlak and Kehat, 2021). Previous study has reported that DUSP6 may inhibit ERK1/2 (Ramkissoon et al., 2019). Based on these theories, we investigated the myocardial expression of DUSP6. As we speculated, Western blot showed that the DUSP6 expression in the DM group was significantly lower than that in the CON group, whereas it was significantly upregulated after SG (Figures 6F,G). The real-time PCR results were consistent with the protein expression results (Figure 6H). These data suggest that the upregulation of DUSP6 may correlate with SG.

### DUSP6 Promotes ERK1/2 Dephosphorylation

To determine the effect of DUSP6 on ERK1/2 dephosphorylation, immunohistochemistry and quantitative analysis were performed. Two consecutive paraffin-embedded heart tissue sections were incubated with primary antibodies for DUSP6 and p-ERK1/2. We observed a clear negative correlation between DUSP6 and p-ERK1/2 expression (Figure 6I). These results were consistent with our previous results (Figures 6A–H). Namely, the results indicated that in the heart tissue, DUSP6 promoted ERK1/2 dephosphorylation, thereby partially ameliorating diabetes-induced cardiac hypertrophy.

## Discussion

With the rapid increase in the number of diabetic patients in recent years, increased attention has been paid to the treatment of diabetes and its complications (Zheng et al., 2018). Among these complications, DCM damage has been widely recognized (Kannel et al., 1974; de Simone et al., 2010), which is defined as left ventricular dysfunction in diabetic patients without coronary heart disease and arterial hypertension (Adeghate and Singh, 2014). One of the main pathological features of DCM is cardiac hypertrophy, which can lead to ventricular dilatation, interstitial fibrosis, and eventually heart failure and death (Kehat and Molkentin, 2010). And the prognosis is mostly poor once the cardiac hypertrophy develops into heart failure (Liu et al., 2019). Unfortunately, the conventional medical therapeutic options for diabetes and obesity, consisting of lowering blood glucose, reversing inflammation, reducing oxidative stress, and gene therapy (Dillmann, 2019; Li and Zhou, 2020), are insufficient (Affinati et al., 2019). Moreover, these above options are not universally effective (Affinati et al., 2019). Thus, more effective therapeutic options are urgently required.

Until 1995, bariatric surgery was only considered as a method for weight loss, but then Pories et al. (1995) proposed that bariatric surgery can treat T2DM and its complications. In recent years, bariatric surgery has gradually become the most effective option for treating diabetes and obesity (Affinati et al., 2019). Among the bariatric surgery procedures, SG has become one of the most used procedures (Pucci and Batterham, 2019). Correspondingly, the HFD-STZ-T2DM rodent model is the most used diabetic model because its characteristics are similar to human T2DM (Gheibi et al., 2017). Thus, in this study, we used this model to investigate the therapeutic effects of SG on diabetes-induced cardiac hypertrophy and the associated mechanisms. It has been reported that 12–16weeks of diabetes were enough to induce cardiac hypertrophy in this model (Ti et al., 2011; Huang et al., 2019). Herein, we found that after 16weeks of diabetes, the heart function was significantly damaged. We added healthy controls to determine the real effect of SG on diabetes-induced cardiac hypertrophy, rather than only comparing the SG group with the SHAM group and the DM group. We found that SG significantly reduced body weight, improved hyperglycemia, and reversed insulin resistance, although some rats regained FBG and body weight after SG. Furthermore, damaged heart function, reduced myocardial glucose uptake, larger area of CMs, and severer myocardial fibrosis along with abnormal expression of cardiac hypertrophy markers were found in our DM model rats. Interestingly, these effects were alleviated to various degrees after SG. Taken together, these results showed that SG alleviated diabetes-induced cardiac hypertrophy.

Diabetes-induced cardiac hypertrophy, which is one of the important manifestations of DCM, is an adaptive response of the heart to many kinds of pathological factors (Jia et al., 2018). Its pathological process can be divided into three stages: the progressive stage, the compensatory stage, and the decompensated stage (Goldenberg et al., 2019). Furthermore, it is mainly manifested as CM hypertrophy, myocardial fibrosis, and increased heart weight (Kobayashi and Liang, 2015). Herein, all the above parameters were significantly improved after SG. These results suggest that SG markedly reversed the damage of myocardial morphology. Notably, the heart function improved to a certain extent as assessed by echocardiography. These results are consistent Sassi et al. (2017). The heart function recovered to a certain extent after the improvement in myocardial morphology, providing direct evidence that SG improved myocardial hypertrophy both in terms of morphology and function.

Myocardial glucose uptake has been reported to trigger DCM and diabetes-induced cardiac hypertrophy (Shao and Tian, 2015). Similar to the fetal heart, the hypertrophic heart has more glucose to fuel metabolism (Shao and Tian, 2015). The major changes in glucose metabolism during cardiac hypertrophy are accelerated glycolysis and downregulated fatty acid oxidation, which means that the myocardial glucose uptake may be reduced (Kolwicz and Tian, 2011). Thus, we performed a PET scan on the hearts of the rats, and SUV was used to evaluate the myocardial glucose uptake. The PET scan results showed a markedly elevated SUV in the SG group, but it did not reach the level of the CON group. These results are consistent with a previous study (Huang et al., 2019). And confirming that SG significantly reverse the myocardial glucose uptake in diabetic and obese rats.

The MAPK signaling pathway (also known as the RAS-RAF-MEK-ERK pathway) is widely involved in the regulation of transcription, differentiation, growth, apoptosis, and movement (Miller et al., 2020). MAPKs mainly comprise p38 MAPKs, JNKs, and ERK1/2. These pathways have a cascade of phosphorylation events of at least three levels: a MAPKK kinase (MAPKKK), a MAPK kinase (MAPKK), and a MAPK (Hatzivassiliou et al., 2010). RAS, a small GTP-binding protein, is the first to activate MAPKKK, which in turn phosphorylates and activates MAPKK, which then activates MAPK (Cargnello and Roux, 2011). MAPK activation mainly depends on phosphorylation of both threonine and tyrosine residues (Cargnello and Roux, 2011). The MAPK signaling pathways have been demonstrated to promote the development of pathological cardiac hypertrophy by regulating cell proliferation, differentiation, apoptosis, growth, and metabolism (Liu and Molkentin, 2016; Liao et al., 2019). Particularly, ERK1/2 plays a key role in pathological cardiac hypertrophy (Lu et al., 2019), because it has been shown to significantly regulate individual CM growth, cardiac dilation, and eccentric growth of the heart (Liu and Molkentin, 2016). Cao et al. (2020) have also reported that excessive ERK1/2 activation induced cardiac hypertrophy. A previous study has reported that almost all MAPKs are activated in pathological cardiac hypertrophy (Toischer et al., 2010), and this phenomenon was confirmed in this study. Interestingly, the expression of phosphorylated p38, JNK, and ERK1/2 was significantly reduced after SG. This result suggests that SG ameliorated diabetes-induced cardiac hypertrophy may be associated with the inhibition of MAPK signaling pathways.

DUSPs, which are MAPK phosphatases, can specifically dephosphorylate MAPKs at both the threonine and tyrosine residues (Liu and Molkentin, 2016). So far, multiple studies have demonstrated that DUSPs inhibited MAPK signaling to improve cardiac hypertrophy (Bueno et al., 2001; Auger-Messier et al., 2013; Liu et al., 2016). ERK1/2, the most important factor affecting cardiac hypertrophy, has been shown to be dephosphorylated and inactivated by DUSP6 (Liu and Molkentin, 2016; Ramkissoon et al., 2019). However, loss of DUSP6 has no effect on p38 and JNK. Because no studies have explored the changes in DUSP6 and ERK1/2 after bariatric surgery, we explored whether there is a connection between DUSP6 and ERK1/2. Immunohistochemistry on consecutive paraffin sections showed that DUSP6 and p-ERK1/2 were upregulated and downregulated, respectively, after SG, which is consistent with our Western blot results. Notably, the expression of DUSP6 and p-ERK1/2 exhibit opposite tendencies. These results suggest that DUSP6 ameliorated diabetes-induced cardiac hypertrophy by dephosphorylating ERK1/2, which is consistent with a previous study (Gallo et al., 2019). These results indicated that SG alleviated diabetes-induced cardiac hypertrophy correlated with the upregulation of DUSP6.

However, there are several limitations to our study. Even though bariatric surgery has an excellent effect on blood glucose and body weight, in the long term, in some patients the blood glucose and body weight return to pre-bariatric surgery levels (Seki et al., 2017). Which leads to the state of the diabetes-induced cardiac hypertrophy after the blood glucose and body weight increase again is still unknown. Therefore, we need to increase the follow-up time in the next study and discuss the long-term ameliorating effect of SG on diabetes-induced cardiac hypertrophy. Additionally, it is unclear whether the SG-induced improvement in diabetes-induced cardiac hypertrophy depends on blood glucose or weight loss. Although we think that the amelioration of the diabetes-induced cardiac hypertrophy depends on improvement in blood glucose and body weight, the exact mechanism remains to be further studied. Finally, to further reveal the SG mechanism in the treatment of diabetes-induced cardiac hypertrophy, we need to investigate the effects of other DUSP isoforms on MAPKs other than ERK1/2 *in vivo* and *in vitro*. We ultimately hope to provide a new strategy for the treatment of diabetes-induced cardiac hypertrophy to reduce the mortality of diabetic patients caused by DCM.

**Figure 7:**
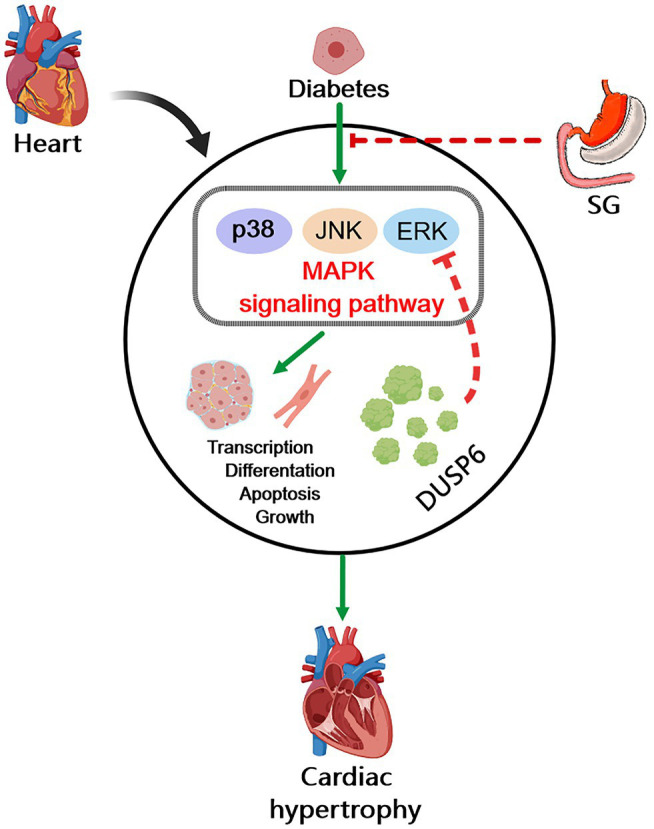
Diagram of possible mechanism of SG improving diabetes-induced cardiac hypertrophy. In diabetes, overactivation of MAPK signaling pathway induces cardiac hypertrophy and heart dysfunction through promoting cell transcription, differentiation, apoptosis, and growth. The inhibition of MAPK signaling pathway is associated with the amelioration of diabetes-induced cardiac hypertrophy by SG. And the dephosphorylation of ERK1/2 by DUSP6 may play a vital role in this possible mechanism.

In conclusion, as a common complication of diabetes, DCM significantly increases the incidence of heart failure in patients with diabetes, thereby severely affecting their life. However, the important pathological manifestation of DCM, cardiac hypertrophy, lacks effective treatment. Our study revealed that diabetes-induced cardiac hypertrophy significantly increased myocardial fibrosis, promoted CM hypertrophy, destroyed the normal myocardial structure, reduced myocardial glucose uptake, and ultimately affected the heart function. Furthermore, the MAPK signaling pathway was activated in diabetes-induced cardiac hypertrophy. Interestingly, all these pathological changes were reversed by SG. Namely, SG attenuated diabetes-induced cardiac hypertrophy correlated with the inhibition of MAPK signaling pathway, particularly, the dephosphorylation of ERK1/2 (Figure 7). In brief, we believe that our study helps resolve some of the challenges of the therapeutic options for DCM, especially diabetes-induced cardiac hypertrophy. Namely, the activation of the MAPK signaling pathways in patients with diabetes-induced cardiac hypertrophy may be a potential target for therapy.

## Data Availability Statement

The raw data supporting the conclusions of this article will be made available by the corresponding author, without undue reservation.

## Ethics Statement

The animal study was reviewed and approved by the Institutional Animal Care and Use Committee of Shandong Provincial Qianfoshan Hospital of Shandong University.

## Author Contributions

GZ and MZ contributed to the original idea and conceptual design. QX, SL, and SD conceived the experiments and analyzed data. QX and HD contributed to the drafting of the work. BS and LL provided critical review of the article. All authors contributed to the article and approved the submitted version.

## Funding

This project was supported by the National Natural Science Foundation of China (Grant No. 81873647) and Major basic research project of Natural Science Foundation of Shandong Province (Grant No. ZR2020ZD15).

## Conflict of Interest

The authors declare that the research was conducted in the absence of any commercial or financial relationships that could be construed as a potential conflict of interest.

## Publisher’s Note

All claims expressed in this article are solely those of the authors and do not necessarily represent those of their affiliated organizations, or those of the publisher, the editors and the reviewers. Any product that may be evaluated in this article, or claim that may be made by its manufacturer, is not guaranteed or endorsed by the publisher.
